# ctDNA-guided precision therapy with trastuzumab deruxtecan plus pyrotinib in HER2-positive breast cancer brain metastases: a case report

**DOI:** 10.3389/fonc.2026.1826447

**Published:** 2026-06-02

**Authors:** Danyang Zhou, Ying Wu, Siyu Guo, Ping Li, Guangxin Li, Mengqi Yang, Shubin Wang, Fang Yang

**Affiliations:** 1 Department of Oncology, Peking University Shenzhen Hospital, Shenzhen, China; 2 Department of Interventional Therapy, Shenzhen Second People’s Hospital, The First Affiliated Hospital of Shenzhen University, Shenzhen, China; 3 Department of Radiotherapy, Peking University Shenzhen Hospital, Shenzhen, China; 4 Department of Pathology, Peking University Shenzhen Hospital, Shenzhen, China; 5 Department of Breast Surgery, Peking University Shenzhen Hospital, Shenzhen, China

**Keywords:** brain metastases, circulating tumor DNA, HER2-positive breast cancer, pyrotinib, trastuzumab deruxtecan

## Abstract

**Background:**

Brain metastases occur in 30-55% of patients with HER2-positive metastatic breast cancer, presenting significant therapeutic challenges. Circulating tumor DNA (ctDNA) monitoring has emerged as a potentially useful tool for earlier molecular detection of disease activity.

**Case presentation:**

We report a 35-year-old woman with HER2-positive metastatic breast cancer diagnosed during pregnancy who achieved pathological complete response after standard therapy. Given her young age, *de novo* stage IV disease, and atypical pregnancy-associated presentation, baseline comprehensive genomic profiling was performed at diagnosis. Serial ctDNA monitoring detected positivity approximately three months prior to radiologic evidence of brain metastases. Genomic profiling revealed newly emergent alterations potentially contributory to evolving disease biology, including MET amplification and HER2 V777L mutation. Treatment with trastuzumab deruxtecan plus pyrotinib, informed by ctDNA dynamics and the molecular profile, was associated with intracranial disease control over 17 months of follow-up. Drug-induced hepatotoxicity was managed through dose modifications and supportive care without treatment interruption.

**Conclusions:**

This case illustrates the feasibility of incorporating ctDNA monitoring into treatment decisions and provides a hypothesis-generating observation regarding combination therapy in managing HER2-positive brain metastases. The temporal relationship between molecular and radiologic findings observed here suggests potential value for earlier detection of disease activity, although whether such lead time translates into improved clinical outcomes requires prospective validation. The findings support prospective evaluation of this approach, including the ongoing TROPHY trial investigating this therapeutic approach.

## Introduction

HER2-positive breast cancer comprises approximately 15-20% of all breast malignancies and shows a high propensity for central nervous system involvement (1, 2). Brain metastases develop in 30-55% of patients with advanced HER2-positive disease, with median survival of 8–18 months despite advances in anti-HER2 therapies (3). The blood-brain barrier poses a formidable obstacle, with most conventional anti-HER2 agents achieving suboptimal central nervous system penetration. Traditional surveillance relies on interval imaging, often detecting progression when tumor burden is substantial and treatment options limited. Furthermore, acquired resistance mechanisms frequently emerge under therapeutic pressure, yet current practice lacks real-time molecular monitoring to guide treatment adaptations. The lag time between disease progression and radiological detection represents a potential interval during which therapeutic options might be reconsidered, although the impact of earlier intervention on outcomes remains to be established.

Circulating tumor DNA (ctDNA) monitoring has emerged as a sensitive method for detecting minimal residual disease and monitoring treatment response, often providing molecular signals of progression before conventional imaging (4). This monitoring capability may inform clinical decisions based on molecular signals in addition to radiological assessment, although whether such monitoring improves patient outcomes requires prospective validation. Beyond early detection, ctDNA profiling reveals tumor genomic evolution under therapeutic pressure, identifying acquired resistance mechanisms that inform treatment selection. In HER2-positive breast cancer, key resistance alterations include MET amplification, which activates bypass signaling pathways and occurs in 15-20% of anti-HER2 resistant cases (5). Additionally, HER2 kinase domain mutations, particularly the V777L mutation, confer resistance to traditional HER2-targeted agents by altering drug binding affinity (6). These molecular insights provided by ctDNA analysis may support consideration of precision treatment approaches targeting specific resistance mechanisms rather than empirical therapy selection.

Recent therapeutic advances offer new hope for brain metastases management. Trastuzumab deruxtecan (T-DXd), an antibody-drug conjugate, has demonstrated activity in brain metastases. The DESTINY-Breast12 trial showed a 62.3% confirmed intracranial objective response rate in patients with active brain metastases (7). Pyrotinib, an irreversible pan-ErbB receptor tyrosine kinase inhibitor, exhibits superior brain penetration compared to other HER2-targeted agents, with a brain-to-plasma ratio of 0.39 versus 0.06 for lapatinib (8).

We present a case illustrating the integration of ctDNA-guided treatment decisions with combination therapy in a patient with HER2-positive breast cancer who developed brain metastases after achieving pathological complete response.

## Case description

### Patient history and diagnosis

A 35-year-old woman presented with a 3.5 cm right breast mass during the 21st week of pregnancy in March 2022. Physical examination revealed axillary lymphadenopathy. Staging workup demonstrated multiple liver metastases on imaging. Core needle biopsy confirmed invasive ductal carcinoma with HER2 positivity (3+ by immunohistochemistry with gene amplification by FISH), estrogen receptor positivity (70%), progesterone receptor positivity (30%), and Ki-67 of 20%. Given her young age, *de novo* stage IV disease, and atypical pregnancy-associated presentation, baseline comprehensive genomic profiling was performed at diagnosis. Genomic profiling using a 520-gene next-generation sequencing panel revealed PIK3CA p.H1047R mutation, TP53 p.R273H mutation, HER2 amplification (copy number 8.2), low tumor mutational burden (4.8 mutations/Mb), and no homologous recombination repair deficiency. Germline BRCA1/2 testing was negative, with no pathogenic variants detected in hereditary breast cancer susceptibility genes.

### Initial treatment and response

Although robust evidence, notably from Amant et al. (9), supports the safety of chemotherapy during the second trimesters, our patient’s *de novo* metastatic HER2-positive disease imposed distinct constraints. Optimal first-line therapy requires dual HER2 blockade with trastuzumab plus pertuzumab—both contraindicated in pregnancy due to oligohydramnios and fetal renal impairment—and durable control depends on prolonged uninterrupted antibody therapy. A multidisciplinary team comprising medical oncology, maternal–fetal medicine, neonatology, psychiatry, and the ethics committee counseled the patient on maternal prognosis without timely anti-HER2 therapy, fetal risks of antibody exposure, and alternative strategies. Recognizing maternal autonomy in pregnancy-associated cancer, she elected to terminate the pregnancy at 21 weeks to permit immediate optimal therapy.

She subsequently received six cycles of docetaxel, trastuzumab, and pertuzumab (THP regimen) with excellent response. After three cycles, the primary breast tumor showed 70% reduction and liver metastases became undetectable on imaging. After 6 cycles of THP regimen chemotherapy, PET-CT demonstrated complete disappearance of detectable lesions in both breast and liver.

While primary tumor surgery is not routine in metastatic breast cancer and recent randomized trials (ECOG-ACRIN E2108, MF07-01) have shown conflicting results, surgery was considered in this case following the exceptional systemic response with complete disappearance of all detectable lesions on PET-CT. Recent evidence suggests that younger patients may derive survival benefit from locoregional treatment, though routine surgery for HER2-positive or oligometastatic disease lacks definitive supportive evidence. This decision was individualized after multidisciplinary discussion and extensive patient counseling.

Right total mastectomy with immediate reconstruction revealed no residual carcinoma, confirming pathological complete response (ypT0 ypN0) in July 2022, providing prognostic information and local disease control. ctDNA testing (OncoMRD-Breast-B, Geneplus-Beijing Co., Ltd., Beijing, China) 4 weeks post-surgery was negative, confirming molecular clearance. Follow-up treatment was initiated in August 2022, consisting of 25 fractions of radiotherapy to the chest wall and regional lymph nodes, concurrently with three cycles of trastuzumab and pertuzumab. The patient then continued the THP regimen until disease progression.

### ctDNA monitoring and recurrent disease

The patient was monitored with serial ctDNA testing and conventional imaging every three months. Twelve months post-surgery, ctDNA testing became positive at 5.7% despite normal imaging. Surveillance frequency was increased. One month later, ctDNA rose to 8.1%; however, imaging still showed no definitive signs of tumor recurrence or metastasis. In this patient, ctDNA positivity preceded radiologic evidence of progression by approximately three months, providing a temporal interval during which clinical reassessment and contingency planning could occur. Treatment for ctDNA-detected progression began in July 2023 with pyrotinib plus capecitabine. However, grade 2–3 diarrhea necessitated treatment modification. Despite treatment modifications, disease progressed, and October 2023 brain MRI revealed multiple brain metastases. The largest lesion measured 13.7×13.9×26.7 mm in the right parietal-occipital region, with additional lesions in bilateral cerebellar hemispheres, parietal and temporal regions. ctDNA levels had increased to 12.3%.

### Genomic evolution analysis

Liquid biopsy genomic analysis at brain metastases detection revealed tumor evolution. While baseline PIK3CA and TP53 mutations persisted, newly emergent alterations included MET amplification and a HER2 V777L kinase domain mutation (2.1% ctDNA fraction). These alterations may be potentially contributory to the evolving disease biology, although their functional significance in this individual patient cannot be definitively established. Notably, HER2 V777L may indicate continued HER2 pathway dependence rather than classical resistance, as kinase domain mutations can retain sensitivity to certain HER2-targeted agents. MET amplification has been associated with bypass signaling in some anti-HER2-resistant cases, though its functional significance in this individual patient cannot be definitively established.

### Combination therapy with T-DXd plus pyrotinib

Based on genomic profiling, T-DXd efficacy in brain metastases, pyrotinib’s brain penetration characteristics, and the potentially contributory genomic alterations described above, combination treatment with trastuzumab deruxtecan (5.4 mg/kg intravenously every 3 weeks) plus pyrotinib (320 mg daily orally) was initiated in October 2023.

The combination rationale was based on complementary mechanisms: T-DXd targeting HER2 through antibody-drug conjugate action and pyrotinib providing broad ErbB receptor inhibition with superior brain penetration. The PIK3CA mutation, present in 35% of HER2-positive breast cancers, has been associated with anti-HER2 resistance, providing additional context for considering combination over sequential therapy. MET amplification raised consideration of dual HER2 blockade to address potential bypass signaling.

### Treatment response and disease control

#### ctDNA monitoring provided molecular insights throughout treatment

**Figure 1:**
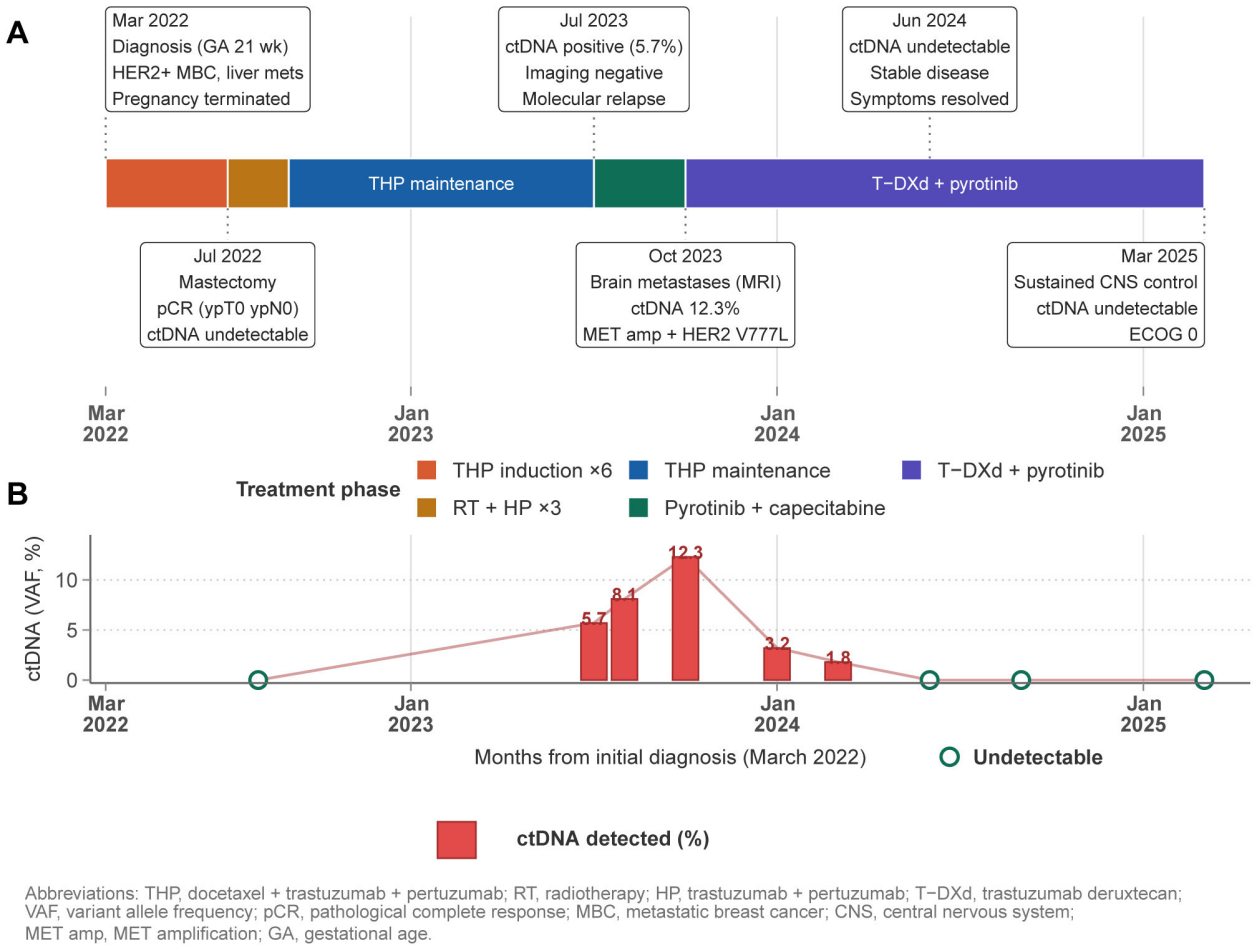
Clinical timeline and serial ctDNA dynamics. **(A)** Chronological overview of treatment phases and key clinical milestones from initial diagnosis (March 2022) through 17 months of sustained intracranial disease control (March 2025). Colored horizontal bars represent five sequential treatment phases: THP induction (docetaxel, trastuzumab, and pertuzumab, six cycles; orange), radiotherapy plus trastuzumab and pertuzumab (RT + HP, three cycles; amber), THP maintenance (blue), pyrotinib plus capecitabine (teal), and trastuzumab deruxtecan plus pyrotinib (purple). Annotated events above the timeline indicate molecular and imaging milestones; annotations below indicate surgical and disease progression events. **(B)** Serial ctDNA measurements plotted as variant allele frequency (VAF, %) against time. Red bars represent timepoints with detectable ctDNA; open circles indicate undetectable ctDNA. The connecting line illustrates the temporal trajectory of tumor-derived circulating DNA from initial molecular relapse through sustained molecular clearance. Vertical dashed lines in both panels denote calendar year boundaries (January 2023, 2024, and 2025).

**Figure 2:**
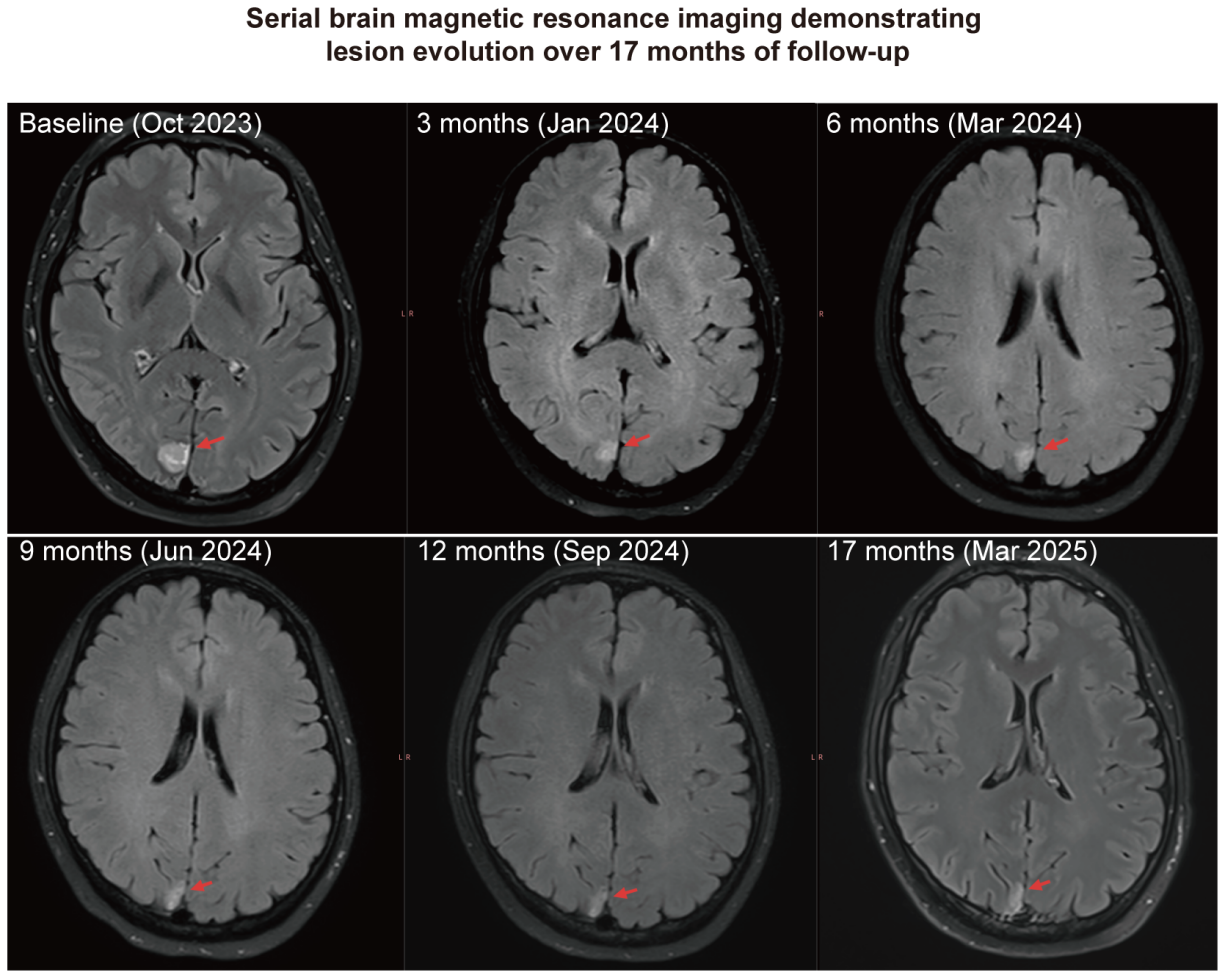
Serial brain magnetic resonance imaging demonstrating lesion evolution over 17 months. Representative axial T1-weighted contrast-enhanced brain MRI sequences from baseline (October 2023) to 17-month follow-up (March 2025). Red arrows indicate the dominant target lesion. Images demonstrate sustained intracranial disease control.

**Figure 3:**
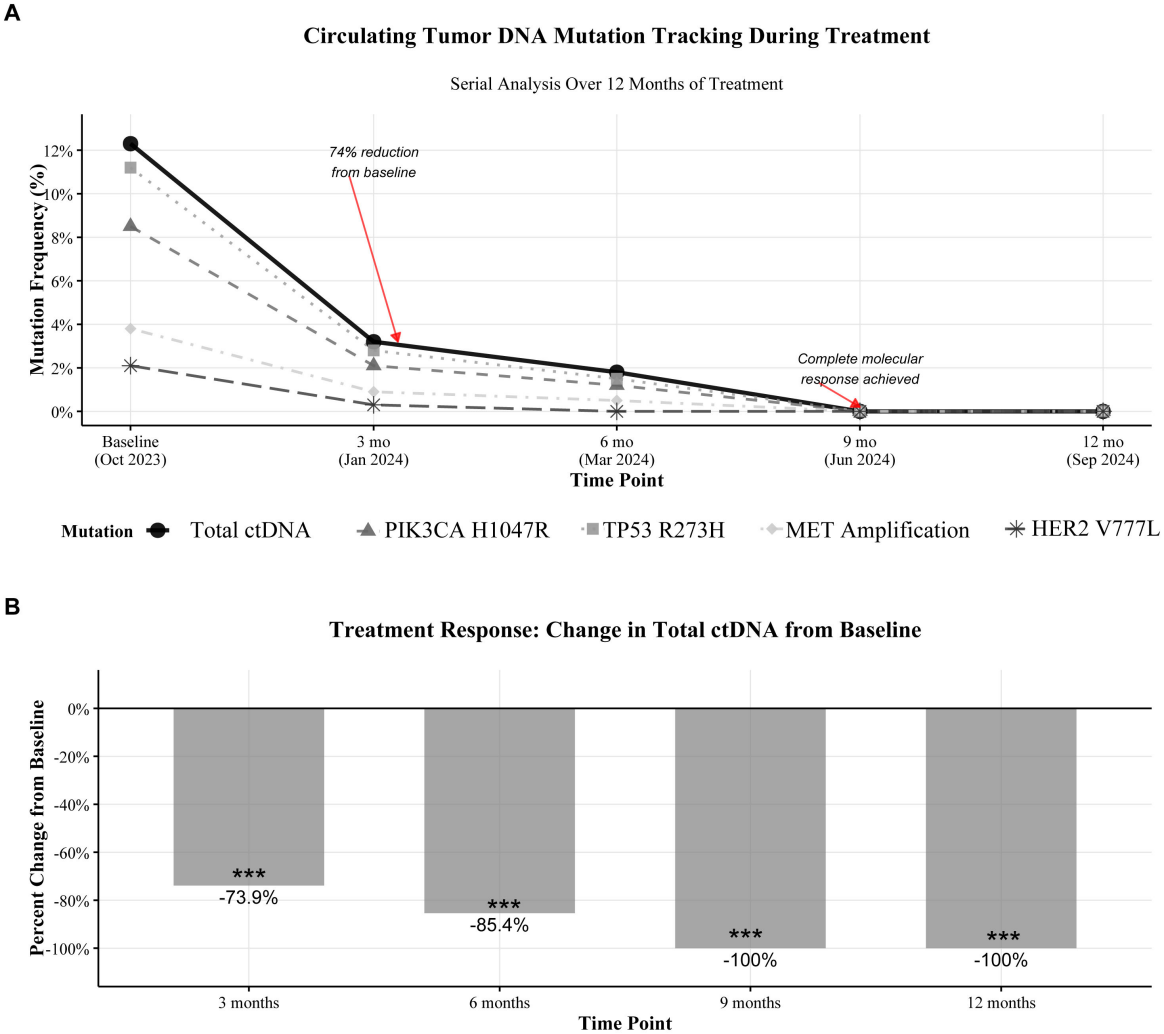
Circulating tumor DNA mutation tracking during treatment. **(A)** Temporal evolution of total ctDNA and individual mutation allele frequencies. **(B)** Treatment response: Change in total ctDNA from baseline.

1. 3 months (January 2024): Brain imaging showed partial response with the largest lesion reduced to 10×9×19 mm. Complete response was maintained for systemic disease. ctDNA decreased to 3.2%, indicating molecular response.
2. 6 months (March 2024): Brain imaging showed mixed response with the largest lesion measuring 12×8×25 mm and new small lesions. Systemic disease remained in complete response. ctDNA further decreased to 1.8%, suggesting continued molecular benefit despite mixed radiological findings.
3. 9 months (June 2024): Disease remained stable with minimal lesion changes and complete resolution of neurological symptoms. ctDNA became undetectable, suggesting a marked reduction in circulating tumor-derived DNA to below the assay detection threshold.
4. 12 months (September 2024) and 17 months (March 2025): Disease control continued with ongoing stability and sustained undetectable ctDNA levels. The patient maintained excellent performance status (ECOG 0) (**Figures 1**–**3**, **Supplementary Figure 1**).

### Toxicity management

The combination was associated with manageable toxicity, primarily hepatotoxicity requiring monitoring and intervention. Initial THP treatment had caused transient liver enzyme elevation (ALT 84 U/L, ALP 175 U/L) that resolved with supportive care.

During the combination therapy with T-DXd Plus Pyrotinib, cholestatic hepatotoxicity was observed, with gamma-glutamyl transferase (GGT) increasing from 25 to 121 U/L and total bile acids increasing from 4.1 to 30.5 μmol/L (**Supplementary Figure 2**).

#### Management strategies enabled continued treatment

1. Enhanced monitoring: Weekly liver function tests, focusing on cholestatic patterns (elevated GGT, alkaline phosphatase, total bile acids).
2. Dose modification: T-DXd interval was extended from every 3 weeks to every 4 weeks rather than discontinuing effective therapy.
3. Hepatoprotective therapy: Early initiation of glycyrrhizin, ursodeoxycholic acid, and S-adenosyl methionine.
4. Multidisciplinary care: Collaboration between oncology and hepatology specialists.

Liver MRI in March 2025 confirmed drug-induced liver injury with early fibrotic changes but demonstrated treatment feasibility. No treatment interruptions were required, allowing sustained therapeutic benefit throughout the 17-month treatment period.

## Discussion

### Clinical impact of ctDNA monitoring

This case suggests the potential utility of molecular surveillance in oncology by illustrating ctDNA’s capacity to detect molecular recurrence three months before radiological evidence. This temporal interval may create therapeutic windows for intervention; whether such lead time translates into improved clinical outcomes requires prospective validation in larger cohorts, particularly in brain metastases where early intervention might prevent neurological compromise and preserve quality of life. The clinical relevance of this molecular surveillance becomes more apparent when its lead time is compared to that of other molecular tests.

Recent studies have begun establishing the predictive value of ctDNA monitoring in metastatic breast cancer, though data specific to HER2-positive brain metastases remains limited. Ma et al. demonstrated that ctDNA-based molecular tumor burden index could detect disease progression 8–16 weeks earlier than conventional imaging in HER2-positive metastatic breast cancer patients, with strong correlation between molecular burden and tumor size (10). The lead time observed in our patient is consistent with this range. Similarly, Coombes et al. reported that ctDNA detection could identify molecular relapse months ahead of clinical relapse in patients with recurrent and metastatic breast cancer, with 89% sensitivity and 100% specificity (11). Whether the magnitude of lead time observed here is reproducible in other patients, assays, or monitoring intervals, and whether it carries clinical actionability, remains to be determined in prospective studies.

The sustained molecular clearance observed throughout treatment provides supportive evidence of therapeutic response when conventional imaging yields ambiguous results. This finding gains additional clinical relevance when considered alongside recent insights into tumor heterogeneity assessment via ctDNA. Ma et al. demonstrated that patients with high tumor heterogeneity detected through ctDNA analysis had significantly worse progression-free survival outcomes compared to those with low heterogeneity (median PFS: 30.0 vs. 60.0 weeks, HR 2.9, p = 0.02). Moreover, the pattern of resistance mutations proved prognostically significant, with patients harboring trunk resistance mutations showing substantially worse outcomes than those with branch mutations (median PFS: 7.8 vs. 27.4 weeks, HR 4.5, p = 0.03) (10). These findings emphasize that detailed molecular profiling through ctDNA monitoring may provide prognostic information beyond simple detection of circulating tumor material.

Furthermore, the detection of newly emergent alterations in our case—specifically MET amplification and HER2 V777L—informed consideration of combination therapy targeting potentially relevant pathways rather than empirical treatment choices. This real-time genomic profiling capability illustrates the feasibility of integrating molecular monitoring into treatment decisions, supporting treatment adaptation based on evolving tumor biology rather than waiting for radiological progression. Whether such treatment adaptation, when guided by molecular monitoring, leads to better outcomes than radiology-driven adaptation alone is an empirical question that this single case cannot resolve. Interestingly, our case demonstrates readily detectable ctDNA levels during brain metastasis progression, which contrasts with recent observations suggesting variable detection rates in isolated brain metastases. Palmieri et al. reported that patients with progressive brain metastases but stable systemic disease showed significantly lower variant allele fractions and fewer somatic alterations compared to patients with active systemic disease (12). This observation underscores the methodological interest of detecting molecular progression in our case, as it suggests either concurrent systemic disease activity or particularly sensitive detection methods capable of capturing brain metastasis-derived circulating DNA.

Importantly, this case illustrates one example of precision medicine principles applied in routine oncology practice. The molecular monitoring approach addresses a recurrent clinical challenge: distinguishing treatment response from progression in the complex radiological landscape of treated brain metastases, where imaging interpretation can be confounded by treatment effects, radiation changes, and pseudo progression. The observed association between undetectable ctDNA and durable clinical control warrants further investigation regarding potential applications in treatment decision-making that extend beyond conventional response criteria. Rather than reactive treatment based on radiological progression, molecular monitoring in this case illustrates the feasibility of proactive therapeutic optimization—an approach that may merit broader evaluation as liquid biopsy technologies mature and accumulate consistent clinical evidence across diverse patient populations. Crucially, the molecular insights provided by ctDNA monitoring in this case did not function as standalone diagnostic information; they directly shaped the combination therapy strategy detailed in the following section, illustrating that molecular surveillance and biomarker-guided treatment selection are operationally inseparable in precision oncology practice.

### Combination therapy efficacy and TROPHY trial validation

This case represents a hypothesis-generating clinical observation regarding T-DXd plus pyrotinib combination therapy, providing real-world experience that may complement the design of the TROPHY trial. The treatment rationale was driven by genomic profiling that revealed a complex, multi-pathway alteration landscape that informed consideration of comprehensive therapeutic intervention rather than sequential single-agent approaches.

The biological rationale for this combination stems from complementary mechanisms of action that address potentially complementary aspects of HER2-driven biology. T-DXd delivers targeted cytotoxic payload through HER2-mediated internalization while potentially disrupting blood-brain barrier integrity, thereby enhancing central nervous system drug penetration. Pyrotinib provides irreversible pan-ErbB inhibition with superior brain pharmacokinetics, with potential activity against kinase domain alterations such as V777L, which may retain sensitivity to certain HER2-directed agents rather than necessarily reflecting classical resistance. This dual approach may provide complementary anti-tumor activity, although the relative contribution of each agent in the setting of these molecular alterations cannot be definitively established from a single case.

The decision to reintroduce pyrotinib in combination with T-DXd, despite earlier progression on a pyrotinib-containing regimen, was supported by a multifactorial rationale. First, central nervous system penetration was a clinically dominant consideration: pyrotinib has a brain-to-plasma ratio of 0.39, substantially exceeding that of lapatinib (0.06), and the PERMEATE trial reported intracranial objective response rates of 74.6% in radiotherapy-naive patients and 42.1% in those with progressive lesions after prior radiotherapy, establishing meaningful CNS activity for pyrotinib-based regimens. Second, the prior progression event warranted re-examination: it occurred on pyrotinib plus capecitabine rather than on pyrotinib monotherapy, manifested predominantly as CNS escape against a background of relatively controlled systemic disease, and was most consistent with capecitabine—rather than pyrotinib—being the limiting therapeutic partner given capecitabine’s limited blood-brain barrier penetration. Pyrotinib was therefore not “failed” in a comprehensive pharmacologic sense, and its residual systemic activity was likely partially preserved. Third, the newly detected HER2 V777L kinase domain mutation provided a distinct mechanistic argument for retained pyrotinib sensitivity, as kinase domain alterations of this class can remain susceptible to irreversible pan-ErbB inhibitors such as pyrotinib and neratinib, which form covalent adducts with the receptor and are less affected by changes in noncovalent binding affinity.

The clinical course observed in this patient under patient-specific, biomarker-driven treatment selection supports prospective evaluation of biomarker-driven combination strategies in precision medicine. Rather than empirical combination therapy, genomic profiling enabled biomarker-informed selection of complementary agents targeting potentially relevant pathways. This case illustrates how individual patient molecular profiling can inform therapeutic approaches that may subsequently inform formal clinical trial development.

The favorable disease control observed in this challenging clinical scenario aligns with a treatment rationale that is now being formally evaluated in the TROPHY trial, which is investigating T-DXd plus pyrotinib in HER2-positive breast cancer with brain metastases. The clinical course described here is consistent with the biological hypothesis underlying that trial and may contribute to the body of real-world evidence informing its interpretation. Importantly, the clinical course observed here would not have been interpretable in the same way without the prior ctDNA-guided identification of MET amplification and HER2 V777L described above; conversely, those molecular findings would have remained an academic observation without a viable biomarker-matched combination to deploy. The two components are therefore most accurately understood as a single integrated precision oncology strategy rather than as independent tools.

An important alternative interpretation must be acknowledged: given the established intracranial and systemic activity of T-DXd monotherapy demonstrated in DESTINY-Breast12 and other trials, it is plausible that much of the observed benefit in this patient was attributable to T-DXd alone, with pyrotinib’s contribution remaining uncertain. This case cannot disentangle the relative contributions of each agent, and prospective trials such as TROPHY are required to establish whether the combination provides incremental benefit over T-DXd monotherapy.

### Toxicity management framework

The hepatotoxicity observed in this case provides important insights into the toxicity profile of T-DXd plus pyrotinib combination therapy. The initial hepatotoxicity during THP therapy (ALT 84 U/L, ALP 175 U/L) was transient and resolved with standard supportive care. However, the hepatotoxicity that emerged during combination therapy showed a different pattern, with progressive elevation of cholestatic enzymes (GGT rising from 25 to 121 U/L, total bile acids from 4.1 to 30.5 µmol/L) suggesting a cholestatic rather than hepatocellular pattern of injury. The imaging correlation showing evolution of hepatic changes provides additional validation of the drug-induced nature of the liver injury.

The successful management strategy employed in this case offers practical guidance for clinicians considering this combination. Weekly liver function monitoring during initial treatment cycles enabled early detection before severe hepatotoxicity developed. The implementation of comprehensive hepatoprotective therapy—including glycyrrhizin, ursodeoxycholic acid, and S-adenosyl methionine—proved effective in controlling liver enzyme elevation while allowing treatment continuation. Importantly, dose interval extension for T-DXd from every 3 weeks to every 4 weeks provided an effective compromise between maintaining therapeutic efficacy and reducing hepatic stress.

The integration of hepatology consultation and liver imaging correlation was crucial for confirming drug-induced liver injury and ruling out disease progression or other hepatic pathology. This multidisciplinary approach enabled evidence-based decision-making regarding treatment continuation versus modification.

For future clinical application of this combination, this case highlights the potential value of enhanced hepatic monitoring protocols, early hepatoprotective intervention, and dose modification strategies. The experience suggests that this combination may be administered safely when appropriate monitoring infrastructure and supportive care measures are in place, offering preliminary guidance for clinical implementation while TROPHY trial data mature.

### Implementation challenges and future directions

While this case describes a favorable clinical course, broader implementation faces significant practical challenges. Successful integration requires reliable access to comprehensive genomic profiling, serial liquid biopsy testing, and multidisciplinary expertise in interpreting molecular data—resources that may not be universally available across practice settings. At our institution, serial ctDNA monitoring has been progressively implemented for high-risk metastatic breast cancer patients since 2021, though we acknowledge this remains investigational and is not yet standard practice internationally. Critical questions remain for prospective validation. Optimal ctDNA monitoring intervals, intervention thresholds, and patient selection criteria for combination approaches require systematic investigation.

The development of standardized toxicity management protocols for novel combinations will be essential for safe clinical implementation beyond specialized centers. The experience also highlights the evolving role of precision medicine in routine oncology practice. This case illustrates feasibility beyond academic medical centers, yet successful integration necessitates careful consideration of resource allocation and care coordination infrastructure in different practice environments. Looking forward, this case highlights key research priorities: prospective validation of ctDNA-guided treatment algorithms, optimization of combination therapy approaches, and development of standardized implementation protocols. While the TROPHY trial will provide formal efficacy and safety data, broader questions about practical implementation in diverse clinical settings remain to be addressed.

## Data Availability

The original contributions presented in the study are included in the article/**Supplementary Material**. Further inquiries can be directed to the corresponding author.

## Supplementary material

Supplementary Figure 1 Comprehensive radiological response analysis integrating tumor volume, molecular markers, and response duration. A. Timeline of target lesion volume and ctDNA levels. B. Percent change in target lesion volume. C. Swimmer plot of response duration. D. Concordance between radiological and molecular response.

Supplementary Figure 2 Hepatic function parameters during combination therapy. Serial analysis of key liver function markers (ALT, AST, ALP, GGT, TBA) over 9 months of therapy.
