# Supplementary figures and images for: ctDNA-guided precision therapy with trastuzumab deruxtecan plus pyrotinib in HER2-positive breast cancer brain metastases: a case report
Source: Front Oncol. 2026 Jun 2;16:1826447. doi: 10.3389/fonc.2026.1826447 (PMC13268879; doi:10.3389/fonc.2026.1826447)

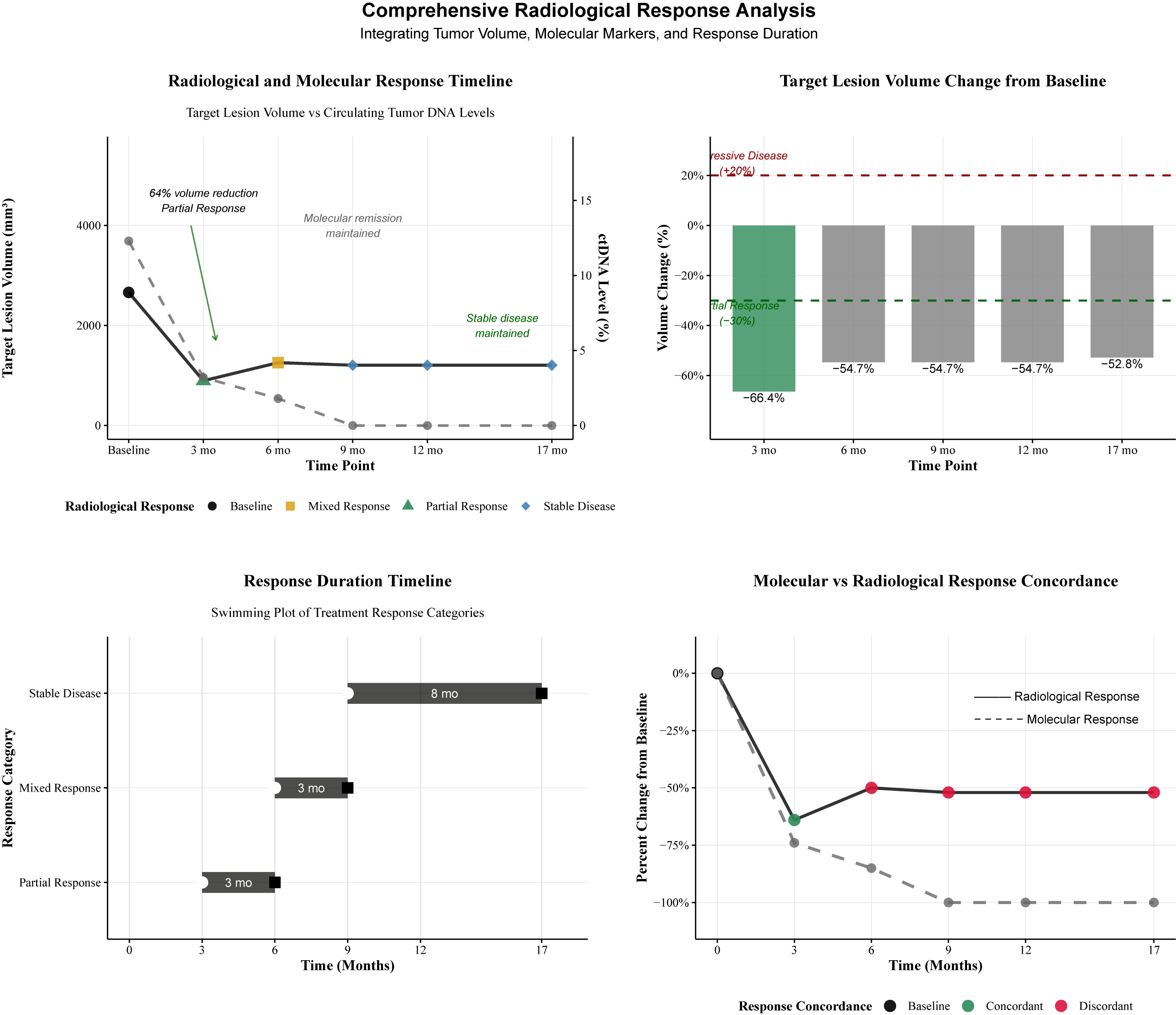

Supplement: Supplementary Figure 1 — Comprehensive radiological response analysis integrating tumor volume, molecular markers, and response duration. A. Timeline of target lesion volume and ctDNA levels. B. Percent change in target lesion volume. C. Swimmer plot of response duration. D. Concordance between radiological and molecular response. [file Image1.tif]

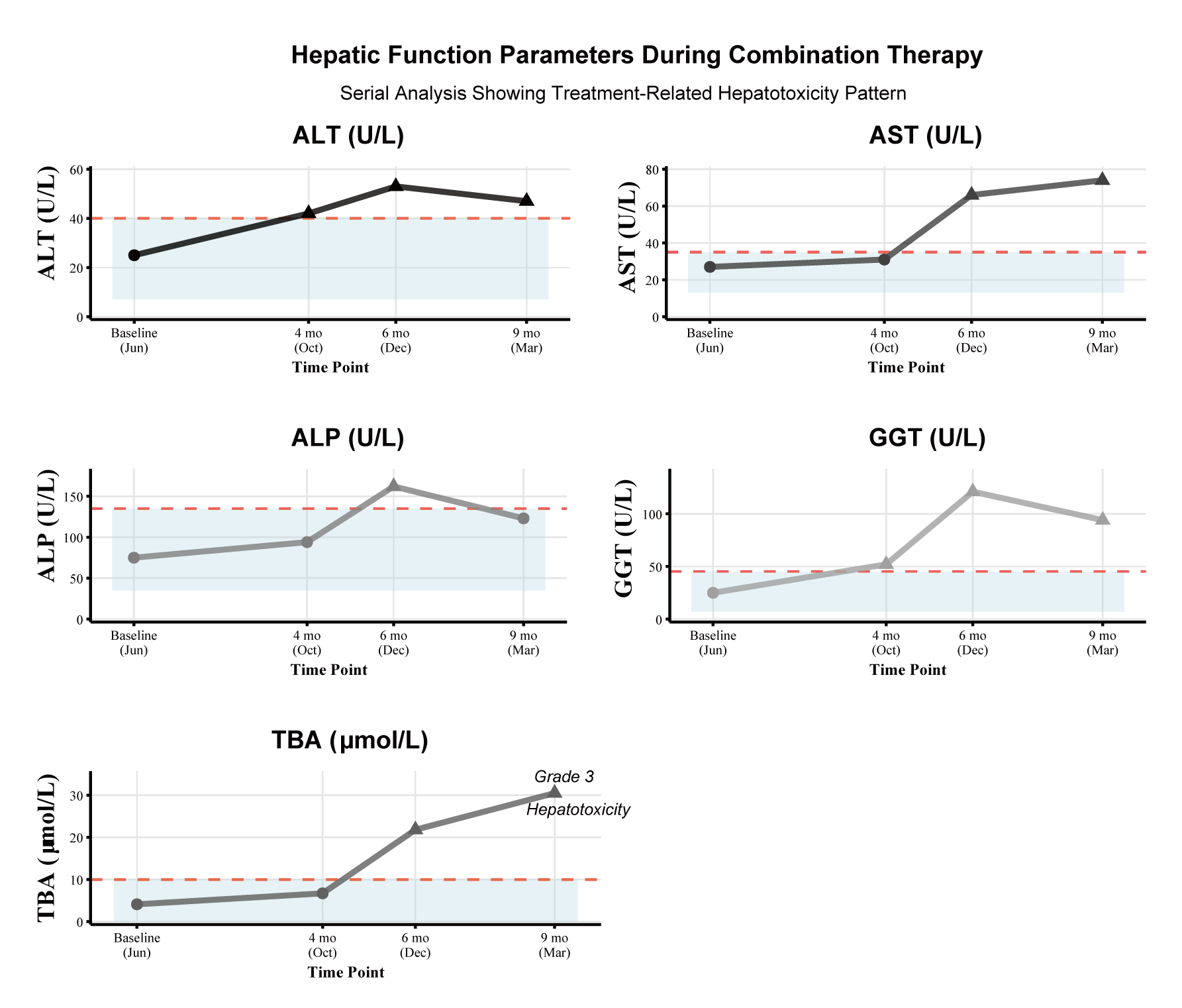

Supplement: Supplementary Figure 2 — Hepatic function parameters during combination therapy. Serial analysis of key liver function markers (ALT, AST, ALP, GGT, TBA) over 9 months of therapy. [file Image2.tif]
